# Does the population size of a city matter to its older adults’ self-rated health? Results of China data analysis

**DOI:** 10.3389/fpubh.2024.1333961

**Published:** 2024-02-01

**Authors:** Zehan Pan, Weizhen Dong, Zuyu Huang

**Affiliations:** ^1^School of Social Development and Public Policy, Fudan University, Shanghai, China; ^2^Department of Sociology and Legal Studies, University of Waterloo, Waterloo, ON, Canada; ^3^School of Public Administration, Hunan University, Changsha, China

**Keywords:** city population size, self-rated health, older adults, sorting decisions, health disparity

## Abstract

Clarifying the association between city population size and older adults’ health is vital in understanding the health disparity across different cities in China. Using a nationally representative dataset, this study employed Multilevel Mixed-effects Probit regression models and Sorting Analysis to elucidate this association, taking into account the sorting decisions made by older adults. The main results of the study include: (1) The association between city population size and the self-rated health of older adults shifts from a positive linear to an inverted U-shaped relationship once individual socioeconomic status is controlled for; the socioeconomic development of cities, intertwined with the growth of their populations, plays a pivotal role in yielding health benefits. (2) There is a sorting effect in older adults’ residential decisions; compared to cities with over 5 million residents, unobserved factors result in smaller cities hosting more less-healthy older adults, which may cause overestimation of health benefits in cities with greater population size. (3) The evolving socioeconomic and human-made environment resulting from urban population growth introduces health risks for migratory older adults but yields benefits for those with local resident status who are male, aged over 70, and have lower living standards and socioeconomic status. And (4) The sorting effects are more pronounced among older adults with greater resources supporting their mobility or those without permanent local resident status. Thus, policymakers should adapt planning and development strategies to consider the intricate relationship between city population size and the health of older adults.

## 1 Introduction

China’s pace of urban development has been notably accelerated in the past three decades. Concurrently, the country has transitioned into an emerging aging society, with 18.9% of its population aged 60 and over by the end of 2021 (1). These intertwined trends have led to an increasing number of older Chinese individuals residing in cities. From 2010 to 2020, the proportion of older Chinese adults living in urban areas rose from approximately 44% to almost 54% (2). Against this background, the quality of life for urban older adults, particularly regarding their health, is gaining heightened attention from both the general public and policymakers in China (3). On one hand, China’s urban landscape is characterized by a hierarchical city system, marked notably by variations in population size. On the other hand, the regional health disparity among older adults tends to expand over the past decade (4). However, no study has yet endeavored to elucidate the association between city population size and older adults’ general health. Addressing this complex yet meaningful question not only aids in comprehending the intercity disparity in older adults’ general health but is also vital for developing targeted strategies to ensure that various cities are age-friendly.

Utilizing data from the micro-data sample of the Chinese 1% national population sample survey in 2015, this study aims to explore the following research questions: Is there an association between city population size and older adults’ health? Does this association differ across various groups of older adults? Do the sorting decisions of older adults regarding residential places alter this association? Multilevel Mixed-effects Probit regression models are constructed to investigate the association between city population size and the self-rated health of urban older adults. Building upon the regression results, we compare the regression residuals between cities of different population sizes to examine older adults’ sorting decisions. Subsequently, a heterogeneity analysis across groups of elders with different characteristics is conducted.

## 2 Literature review

### 2.1 Urban population growth in China’s cities

Since the gradual relaxation of population movement restrictions in the 1980s, surplus agricultural laborers in rural China began migrating to cities in pursuit of non-farm wage employment opportunities, marking the initiation of China’s rapid urbanization process. While many rural–urban migrants initially settled in cities near their hometowns, a considerable number undertook long-distance migration to the country’s major urban centers. Consequently, Chinese cities have undergone substantial population growth over the past three decades, leading to an increase in the number of cities across all population size brackets (5). Notably, the growth in the number of cities with residents exceeding 1 million has outpaced that of smaller cities. The central and western regions have witnessed a more pronounced increase in the number of cities with populations below 5 million, while the eastern region has experienced a more substantial rise in cities with over 5 million residents. As of 2020, the average population of Chinese cities stood at approximately 0.84 million. Among all cities in China, 522 have populations ranging from 0.1 to 1 million, 84 have populations between 1 and 5 million, and 21 exceed 5 million residents (2). Consequently, China’s urban landscape is characterized by a hierarchical city system, notably marked by variations in population size.

### 2.2 Health disparity among older adults across cities

While numerous studies delve into the rural–urban gap and regional patterns at a broader spatial scale, there is a distinct shortage of investigations examining nuanced intercity variations in general health among older adults. To deduce the potential effects of urban population growth on the general health of older adults, we conducted a review of the determinants of health associated with population growth.

At the individual and household level, it has become evident that disparities in individual socioeconomic status play a predominant role in the health disparity experienced by older adults (6). A decomposition analysis of the concentration index, following the framework outlined by Wagstaff (7), revealed that household income significantly contributes to health inequality among Chinese older adults, accounting for 41.15% (8). This income-related contribution surpasses that of gender or age, underscoring a pervasive health disparity between affluent and economically disadvantaged older populations in China.

On a regional scale, the socioeconomic development level of cities emerges as a pivotal determinant shaping disparity in the general health of older adults across cities with varying population sizes. First, urban population expansion can positively impact residents’ health through economies of scale. Population growth in a city often translates to increased employment opportunities and more secure incomes (9, 10). Second, residents in larger cities are more likely to enjoy enhanced public services due to superior local fiscal conditions. Over the past decades in China, a rapid concentration of the population, particularly the working-age demographic, towards more developed cities such as provincial capitals and metropolitan areas can be observed (11). This population redistribution has resulted in intercity disparities in socioeconomic development and corresponding incongruities in local fiscal conditions. In China, sub-national governments bear the primary responsibilities for public goods and services within the decentralized fiscal system (12). Approximately 47.6% of the total budgetary revenue was collected by the local governments, while they bore 80% of the total budgetary expenditure (13). Hence, the provision of healthcare facilities, services, and health insurance and pension schemes greatly varies among cities, which further influences older adults’ health disparity (14, 15). Cities with larger populations find it easier to provide superior infrastructure and public services in health, sanitation, and education at lower fixed costs (10, 16, 17). Individuals in larger cities are also more likely to benefit from a comprehensive security system (18, 19). According to the Annual Report on Urban Health Life 2021 in China, Beijing and Shanghai hold the top two positions in both overall resident health and health equality among residents (20).

However, urban population growth also has negative impacts on residents’ health. Overcrowded cities are more vulnerable to “urban diseases” such as traffic congestion, environmental degradation, scarcity of natural resources, the decline in living space, and the increase in crime (21–23). The overexpansion of cities is also likely to deteriorate neighborhood conditions that jeopardize urban residents’ health. For instance, people living in relatively poor conditions (in a densely populated area, away from the natural environment) may face difficulties in social relations with others (24, 25). The quality of health-related services received by urban residents would be compromised if the supply growth of community medical resources could not keep up with the relentless growth of the population (10). Similar examples can also be found for other pathways where community poverty, deprivation, and disadvantage occur (26–28). In addition, urban population growth is believed to be associated with reducing urban residents’ physical activity, thus putting them at greater risk of chronic disease. These unfavorable outcomes led by urban expansion, commonly known as the urban health penalty, would mitigate the positive effects of urban expansion on the residents’ health.

### 2.3 Sorting decisions

From an individual standpoint, when individuals evaluate the advantages and disadvantages of residing in densely populated cities, they often make choices based on their health status, adding a layer of complexity to the correlation between a city’s population size and the health of its residents (29, 30). According to the Emigration Selection Theory, individuals contemplating a move may decide on their destination based on their health condition (31–33). Those with poorer health are less inclined to become migrants, primarily due to the physically and/or psychologically demanding nature of jobs typically associated with migration destinations, which tend to select against individuals with adverse health conditions (32). The Salmon Bias Hypothesis also supports this notion, positing that individuals in poor health are more likely to return to or relocate closer to their hometowns than their healthier counterparts. This is attributed to the perception that unhealthy migrants may face increased challenges in the labor market when residing far away from their hometowns (31, 34). Moreover, older adults with diminished health status may encounter greater difficulty adapting to life in larger cities (35–37). Consequently, the potential health benefits associated with urban population growth might be overstated if the impact of these individual sorting decisions is not duly taken into account.

### 2.4 China’s urbanization policy

Given the varying advantages and challenges arising from the surge in urban population, there exists a lack of consensus among policymakers and scholars regarding the most effective urbanization strategy. The China’s 14th Five-Year Plan (2021–2025) advocates for the removal of household registration restrictions in medium and small cities to absorb rural–urban migrants (38). Building on this initiative, in 2022, China’s State Council introduced a policy aimed at advancing urbanization, specifically targeting county towns (39, 40). However, within academic circles, there appears to be a lack of empirical support for this policy. Drawing upon the theory of economies and diseconomies of scale, various studies have unveiled an inverted U-shaped relationship between city population size and key indicators such as labor productivity (41), natural resource utilization efficiency (42), environmental impacts (43), and attractiveness to migrants (44) in Chinese cities. Notably, the turning points identified in these studies far surpass the population upper threshold defining medium and small cities in China. Despite the wealth of research, the growing urban population’s impact on the health of older residents in an increasingly aging China remains inadequately explored.

## 3 Materials and methods

### 3.1 Data and measures

#### 3.1.1 Data

In 2015, China conducted the 1% population sample survey, commonly referred to as the “mini-census” (45). The National Bureau of Statistics has provided access to a micro-level database derived from a random 10% sample of respondents from this survey. Notably, this micro-level database comprises comprehensive information on the health, sociodemographic, and geographical characteristics of the surveyed individuals. It shares both content and organizational structure similarities with a census, providing extensive coverage and a high level of representativeness.^1^

Additionally, relevant data reflecting the characteristics of cities were sourced from various reputable publications, including the China Statistical Yearbook, China City Statistical Yearbook, China Urban Construction Statistical Yearbook, China Public Health Statistical Yearbook, China National Environmental Real-time Monitoring Centre, China National Meteorological Science Data Center, and China National Catalogue Service for Geographic Information. These diverse data sources contribute to a comprehensive understanding of the contextual factors and environmental elements associated with the urban areas under study.

#### 3.1.2 Health indicator

Self-rated health (SRH) serves as a subjective indicator that reflects an individual’s assessment of their physical and mental well-being. Recognizing its significance, the World Health Organization considers self-rated health a crucial metric for population health, and it has been widely employed in various health studies (46, 47). In the 1% population sample survey, respondents were tasked with evaluating their current health status using the question: “How do you rate your current health status: healthy, fairly healthy, unhealthy-can live independently, unhealthy-cannot live independently?”

However, a potential bias may exist, as different respondent groups may interpret and use ordinal response categories differently (48). Specifically, concerning the self-rated health question in this survey, the criteria for reporting “healthy” versus “fairly healthy” show more variability than those for reporting “healthy” versus “unhealthy.” Consequently, we opted to group the first two choices as “healthy” with an assigned value of 1 and categorized the remaining options as “unhealthy” with an assigned value of 0. This approach aims to mitigate potential ambiguity in the interpretation of responses and ensures a clearer distinction for analytical purposes.

#### 3.1.3 Defining “city”

**Figure 1:**
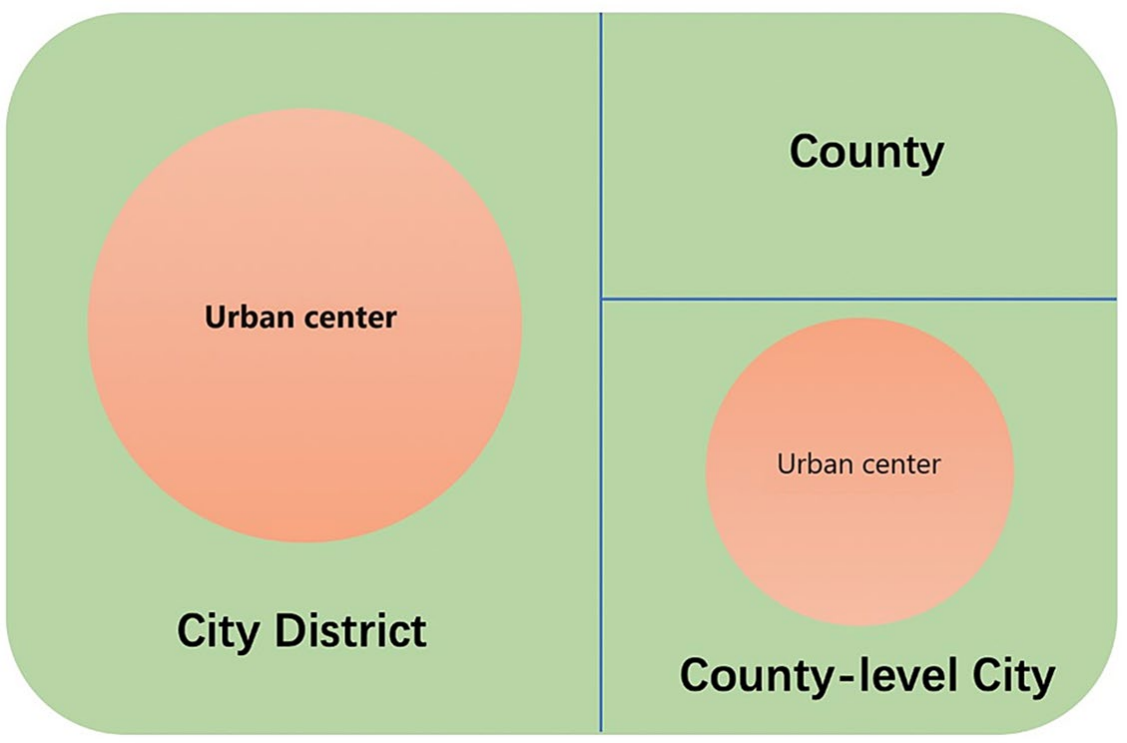
Prefectural-level divisions of Chinese cities.

The term “city” in China is commonly associated with a prefectural-level region rather than a specific urban center. A prefectural-level region typically comprises three distinct administrative divisions: city districts, county-level cities, and counties (see Figure 1). Despite sharing the same administrative level, these divisions have undergone varying degrees of urbanization. City districts exhibit the highest level of urbanization, followed by county-level cities, which encompass both urban and surrounding rural areas. Counties, however, typically feature small towns but generally lack prominent urban centers.

Utilizing urban and rural classification codes at the village level, we identified the adjacent urban areas within each city district or county-level city, designating them as “urban centers” or, equivalently, “cities.” Counties were excluded from its scope. We matched individual data to these designated cities using village-level administrative codes and computed the city population size by aggregating residents who had resided in a city for over six months. The resulting dataset encompasses 79,821 older adults residing in 599 cities, inclusive of inclusive of 307 urban centers in city districts and 292 urban centers in county-level cities, spread across 30 provinces.

Table 1 shows the self-rated health status among older adults in cities of different population sizes. Most respondents from cities with more than 10 million residents reported being “Healthy” and “Fairly healthy” (91.81%). The respondents residing in the cities with smaller population sizes reported slightly less-healthy, and the proportion of the healthy group generally declined as the city size decreased. In the cities with less than 0.1 million residents, 88.95% of respondents reported being healthy or fairly healthy. These observations preliminarily indicate that the city’s population growth may promote the self-rated health of its older adults. However, whether there is a linear association between them needs to be tested.

**Table 1:** Self-rated health of the older adults in cities of different population sizes.

City’s population size (million)	0.1−	0.1–1	1–5	5–10	10+
Mean value of city population size (million)	0.29	0.74	2.27	6.61	18.78
Self-rated health of the urban older adults (%)					
Healthy	88.95	89.27	90.73	91.17	91.81
Unhealthy	11.05	10.73	9.27	8.83	8.19

#### 3.1.4 Covariates

We built upon existing research (16, 49, 50) and employed rigorous controls for a diverse set of individual covariates spanning three key aspects: demographic, living status, and socioeconomic status. Within the demographic realm, variables such as gender, ethnicity, age, and marital status (having a spouse or not) were considered. Living status indicators encompassed whether individuals lived alone, the presence of basic amenities such as toilets and kitchens, and *per capita* living space. The socioeconomic status dimension involved factors like educational attainment, resident registration status, employment status, land ownership (having contracted farmlands or not), sources of income, possession of private cars, participation in pension insurance programs, and enrollment in medical insurance schemes. This comprehensive approach aimed to account for a wide range of individual characteristics that could potentially influence the outcomes under investigation.

At the city level, we initially controlled for variables pertaining to a city’s geographical conditions, encompassing annual precipitation, sunshine duration, annual average temperature, medial humidity, average land relief, average altitude, and the least distance to seaports. These exogenous variables are anticipated to simultaneously influence a city’s population size and residents’ health, without being reciprocally affected.

Further, we controlled for the variables related to the city’s socioeconomic and human-made environment (16, 49–51). These variables are expected to interact with population size and the health of older adults, serving as potential pathways through which a city’s population growth impacts the health of its older residents. Recognizing the potential collinearity among covariates depicting the cities’ socioeconomic and human-made environment, we employed factor analysis to consolidate four principal factors: Socioeconomic Development Index, Environmental Pollution Index, Afforestation Index, and Pollution Abatement Index (Appendix). This combination effectively characterized a city’s contextual characteristics and accounted for approximately 74% of the total variability in the original variables.

### 3.2 Empirical analysis method

#### 3.2.1 Multilevel mixed-effects Probit regression models

We adopted Multilevel Mixed-effects Probit regression models to account for the lack of sample independence within the cities and control the constant differences across the cities. It helps to identify the group structure at both individual and city levels. We included city population size and its quadratic term to explore whether there is a nonlinear association between city population size and the self-rated health of older adults. The following is our proposed Probit model (Equation 1):


(1) $$\begin{array}{l} Prob\left(Health_{ij}\right)=\\ \Phi \left(\beta_{1}Pop_{size}{\;}_{j}+\beta_{2}Pop_{size}{{\;}^{2}}_{j}+\gamma_{1}X_{ij}+\gamma_{2}X_{j}+\varepsilon_{j}\right) \end{array}$$

Where $Health_{ij}$ depicts the self-rated health of the older adults i living in the city *j*, $Pop\_size_{j}$ and $Pop\_size^{2}{\;}_{j}$ are the population size of city *j* where older adult i lives and its quadratic term, respectively. $X_{ij}$ and $X_{j}$ denoted a series of individual and city-level covariates. $\varepsilon_{i}$ is the residual term.

#### 3.2.2 Sorting analysis

While we controlled for various individual and city-level variables, there were still unobserved ones likely to confound with the associations between city population size and the self-rated health of older adults. A useful approach to discerning the impact of these unobserved factors is to compare the residual distribution of self-rated health among cities with varying population sizes (52). The distinctions in the residual distribution offer an illustration of the sorting decisions made by older adults based on the unobserved individual and cities’ characteristics.

Following the sorting method proposed by Combes et al. (52), we identified the shift, dilation, and truncation of the distribution in the self-rated health residuals of older adults living in cities of different sizes. We made $\lambda_{k}\left(\mu \right)$ to be the quantile of the distribution $F_{k}$ at rank μ, and $F_{k}$ is the Cumulative Distribution Function of the self-rated health residuals of older adults with k (1 = smaller cities, 2 = larger cities). The transformation of the self-rated health residuals yields the following relationship between the two distributions (Equation 2):


(2) $$\begin{array}{c} \lambda_{2}\left(\mu \right)=D\lambda_{1}\left[S+\left(1-S\right)\mu \right]+A,\mu \in \left[0,1\right] \end{array}$$

whereA is a shift parameter, D is a dilation parameter, and S is the rank that left truncation to occur.

**Figure 2:**
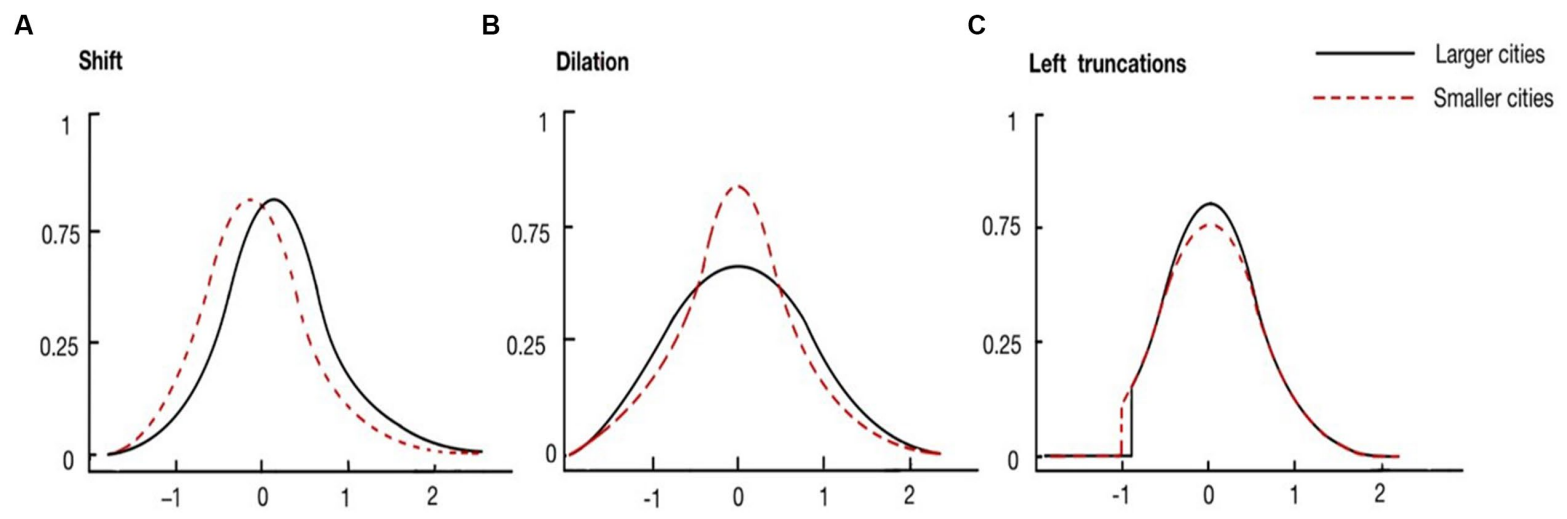
Three possible transformations of the distribution of self-rated health residuals. (From left to right: **(A)** shift, **(B)** dilation, **(C)** left trunctions).

Parameter A could be positive or negative, contingent on whether there was a right or left shift in the distribution of the self-rated health residuals of older adults in larger cities relative to that of smaller ones. A positive shift (A > 0) refers to the positive effects of city population size on older adults’ health. Figure 2A considers the case of a simple positive shift only (A > 0).

Parameter D could be above or below one, indicating whether there was a dilation or compression in the distribution of the residuals of older adults in larger cities relative to that of smaller cities. This parameter detects the difference in the heterogeneity of elders’ self-rated health between cities of different population sizes. Figure 2B considers the case of a simple dilation only (D > 1).

Parameter S compared left truncations between the two distributions. The case of “positive” truncation (S > 0) corresponds to a situation where the distribution in larger cities would be more truncated than in smaller cities. In other words, larger cities have fewer older adults with poorer health status. This parameter examines whether there is a sorting effect for less-healthy older adults. Figure 2C considers the case of a simple left truncation only (S > 0).

## 4 Empirical results

### 4.1 From a linear to an inverted U-shaped relationship: controlling for the individual characteristics

We adopted a stepwise approach to incorporate covariates into the Multilevel Mixed-effects Probit regression models. The initial results, presented in the first two columns in Table 2, depict findings without adjusting for individual and city-level characteristics. In Model 1_1, a notable coefficient of 0.053 for city population size, signifying significance at the 1% level, indicates a positive correlation with the self-rated health of older adults. Introducing the quadratic term in Model 1_2, an insignificantly negative coefficient for the quadratic term emerges at the 10% level, suggesting a linear relationship between city population size and the self-rated health of older adults. Upon incorporating individual characteristics related to demographic and living status into the models, the coefficients for city population size remain significant at the 1% level (Model 2_1, Model 2_3). Additionally, the coefficients for the quadratic terms of city population size continue to be insignificant at the 10% level (Model 2_2, Model 2_4).

**Table 2:** Results of multilevel mixed-effects probit regression models controlling for individual characteristics.

	Model 1_1	Model 1_2	Model 2_1	Model 2_2	Model 2_3	Model 2_4	Model 2_5	Model 2_6	Model 2_7	Model 2_8
Turning point (million people)								1.16		1.52
City population size	0.053^***^ (0.013)	0.130^***^ (0.073)	0.069^***^ (0.014)	0.158^*^ (0.082)	0.048^***^ (0.013)	0.106 (0.072)	0.019 (0.014)	0.218^***^ (0.079)	0.025^*^ (0.015)	0.188^**^ (0.082)
Quadratic term of city population size		−0.009 (0.008)		−0.0102 (0.009)		−0.007 (0.008)		−0.023^***^ (0.009)		−0.019^**^ (0.009)
Demographic	–	–	Yes	Yes	–	–	–	–	Yes	Yes
Living status	–	–	–	–	Yes	Yes	–	–	Yes	Yes
Socioeconomic status	–	–	–	–	–	–	Yes	Yes	Yes	Yes
Individual characteristics	No	No	Partially	Partially	Partially	Partially	Partially	Partially	Yes	Yes
Cities’ characteristics	No	No	No	No	No	No	No	No	No	No
*N*	79,821	79,821	79,821	79,821	79,821	79,821	79,821	79,821	79,821	79,821

(a) Standard errors in parentheses. (b)^*^ *p* < 0.1, ^**^ *p* < 0.05, ^***^ *p* < 0.01.

However, the linear association between city population size and the self-rated health of urban older adults disappears when introducing individual characteristics related to socioeconomic status (Model 2_6). The coefficient of the quadratic term, which is significant at the 1% level, indicates an inverted U-shaped association with a turning point of 1.16 million. Furthermore, when incorporating all individual covariates, the coefficient of the quadratic term, significant at the 5% level, demonstrates an inverted U-shaped association with a turning point of 1.52 million (Model 2_8).

### 4.2 Shifted turning point: controlling for cities’ characteristics

Our exploration of city-level covariates reveals shifts in the optimal population size when accounting for geographical, socioeconomic, and human-made environmental factors (Table 3). Incorporating city-level variables associated with geographical conditions (Model 3), the turning point notably decreases to 1.03 million. In Model 4_1 and 4_3, where Socioeconomic Development Index and Afforestation Index were considered, both exhibited positive associations with the self-rated health of urban older adults. Notably, in comparison with Model 3, the turning point drops to 0.56 million in Model 4_1. Conversely, the Pollution Index emerges as an adverse factor impacting the self-rated health of urban older adults. The turning point increases to 1.12 million in Model 4_2. The Pollution Abatement Index shows no significant association with the health indicator in Model 4_4. This outcome is unsurprising, given that China’s pollution control practices have only gained momentum following the introduction of a stringent environmental policy by the central government in 2015 (53). Finally, the introduction of all factors related to the socioeconomic and human-made environment amplifies the inverted U-shaped association between city population size and the self-rated health of older adults, pinpointing a turning point at 0.47 million residents (Model 4_6). Simultaneously, the linear relationship became imperceptible (Model 4_5).

**Table 3:** Results of multilevel mixed-effects probit regression models controlling for city-level characteristics.

	Model 3	Model 4_1	Model 4_2	Model 4_3	Model 4_4	Model 4_5	Model 4_6
Turning point (million people)	1.03	0.56	1.12		1.06		0.47
City population size	0.131^*^ (0.072)	0.234^***^ (0.091)	0.146^**^ (0.070)	0.106 (0.073)	0.127^*^ (0.071)	−0.002 (0.019)	0.240^***^ (0.091)
Quadratic term of city population size	−0.0141^*^ (0.008)	−0.029^**^ (0.011)	−0.016^*^ (0.008)	−0.012 (0.008)	−0.014^*^ (0.008)		−0.0312^***^ (0.012)
Socioeconomic development index		0.105^*^ (0.057)	–	–	–	0.021 (0.044)	0.140^**^ (0.062)
Environmental pollution index	–	–	−0.034^**^ (0.017)	–	–	−0.024 (0.018)	−0.022 (0.017)
Afforestation index	–	–	–	0.023^*^ (0.013)	–	0.027^**^ (0.013)	0.032^**^ (0.013)
Pollution abatement Index	–	–	–	–	−0.016 (0.015)	−0.016 (0.015)	−0.010 (0.015)
Individual characteristics	Yes	Yes	Yes	Yes	Yes	Yes	Yes
Cities’ socioeconomic and human-made environment	No	Partially	Partially	Partially	Partially	Yes	Yes
Cities’ geographical conditions	Yes	Yes	Yes	Yes	Yes	Yes	Yes
*N*	79,821	79,821	79,821	79,821	79,821	79,821	79,821

(a) Standard errors in parentheses. (b)^*^ *p* < 0.1, ^**^ *p* < 0.05, ^***^ *p* < 0.01.

### 4.3 Sorting effects: small cities host less-healthy older adults

Given the potential influence of unobserved factors on the association between city population size and older adults’ self-rated health, we conducted a comparison of the residuals’ distribution derived from Model 4_6 among cities of varying population sizes using four cut-off points (Table 4). First, negative estimates of Shift A indicate that unobserved factors contribute to a relatively poorer average health status among older adults in larger cities compared to those in smaller cities. Second, positive estimates of Dilation D suggest that self-rated health residuals of older adults in larger cities tend to be more concentrated in the middle range, indicating that unobserved factors contribute to a lower dispersion of health residuals among older adults in larger cities.

**Table 4:** Comparing health residuals of older adults between cities of different population sizes.

	Shift A	Dilation D	Truncation S	*N*	Pseudo *R*^2^
H_0-0.5_ to H_0.5–1_	−0.134^***^ (0.013)	0.911^***^ (0.015)	−0.000 (0.004)	35,039	0.768
H_0-0.5_ to H_1-5_	−0.151^***^ (0.014)	0.891^***^ (0.025)	0.000 (0.012)	42,163	0.6932
H_0-0.5_ to H_5-10_	−0.15^***^ (0.012)	0.937^***^ (0.012)	0.046^***^ (0.005)	27,037	0.654
H_0-0.5_ to H_10+_	−0.349^***^ (0.013)	0.91^***^ (0.011)	0.064^***^ (0.004)	31,043	0.890
H_0.5–1_ to H_1-5_	−0.02 (0.015)	0.977^*^ (0.013)	0.000 (0.003)	40,228	0.375
H_0.5–1_ to H_5-10_	−0.036^*^ (0.019)	0.973 (0.022)	0.016^*^ (0.008)	25,102	0.415
H_0.5–1_ to H_10+_	−0.230^***^ (0.015)	0.954^***^ (0.011)	0.034^***^ (0.004)	29,108	0.882
H_1-5_ to H_5-10_	−0.049^***^ (0.016)	0.945^***^ (0.012)	0.000 (0.003)	32,226	0.608
H_1-5_ to H_10+_	−0.227^***^ (0.014)	0.944^***^ (0.011)	0.018^***^ (0.003)	36,232	0.899
H_5-10_ to H_10+_	−0.195^***^ (0.019)	0.97^*^ (0.016)	0.008^*^ (0.004)	21,106	0.961

(a) H_x-y_: distribution of self-rated health residuals of older adults in cities where no more than y million residents live but more than x million residents live. (b) Standard errors are in parentheses. (d)^*^ *p* < 0.1, ^**^ *p* < 0.05, ^***^ *p* < 0.01.

**Figure 3:**
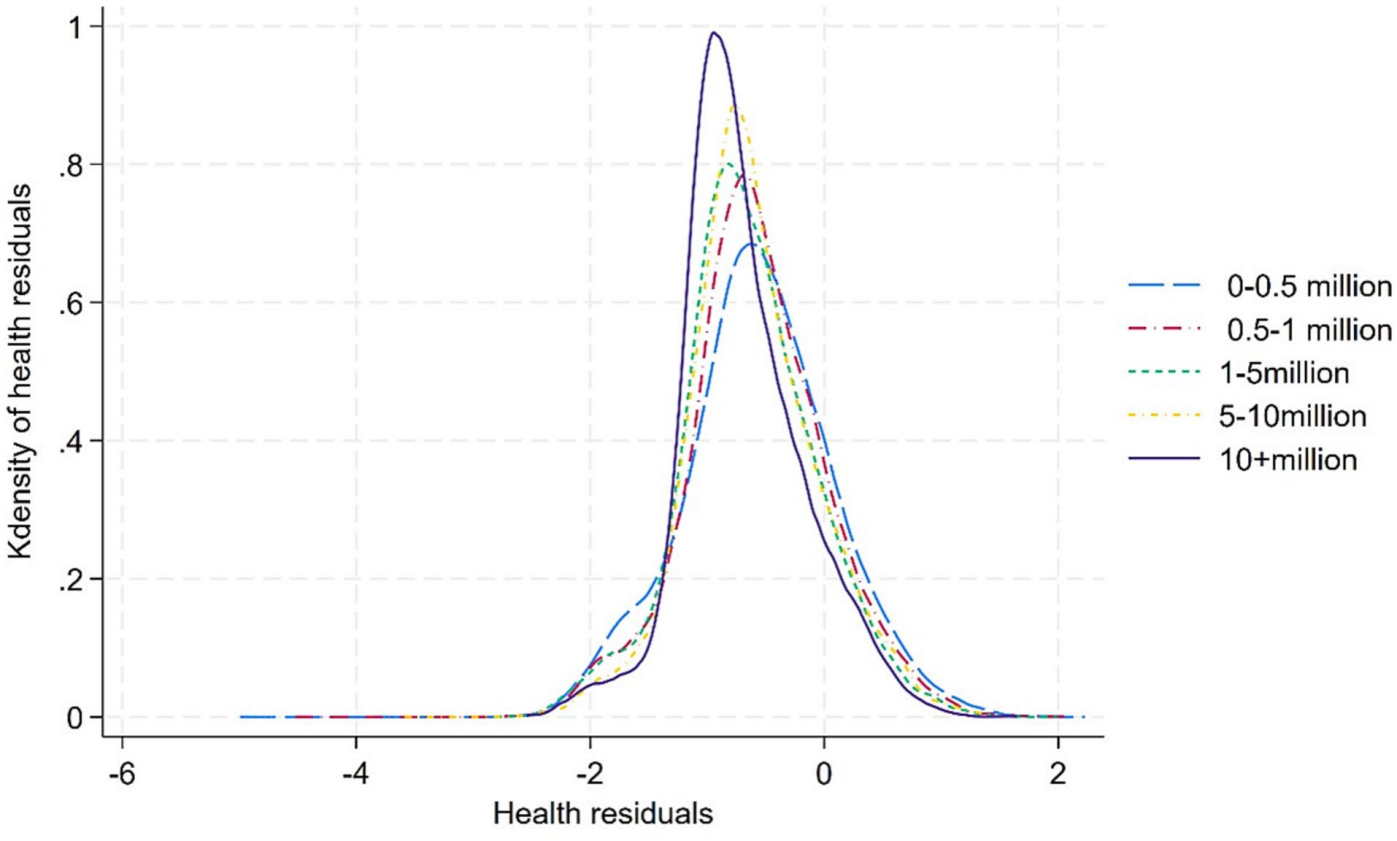
Health residuals of older adults in smaller vs. larger cities.

Third, truncation S estimates are significantly positive in models comparing health residuals between cities with less than 5 million residents and those with more than 5 million residents, but not significant in models comparing health residuals among cities with less than 5 million residents. These findings suggest that unobserved factors tend to concentrate less-healthy older adults in cities with less than 5 million residents. Notably, a larger truncation estimate is observed between cities with a more significant disparity in population size, with the most noticeable statistical difference in truncation parameters found between cities with less than 0.5 million people and those with over 10 million people, as illustrated in Figure 3.

### 4.4 Heterogeneity across different groups of older adults

We examined how the associations between city population size and the self-rated health of older adults varied by individual characteristics, as outlined in Table 5. The relationship between city population size and the self-rated health of older adults exhibits an inverted U-shaped curve for those who were female, aged below 70, not living alone, or had received education at junior high school and above. This pattern persists both before and after accounting for cities’ socioeconomic and human-made environment. For their counterparts, the statistically significant inverted U-shaped associations emerges solely after controlling for the cities’ socioeconomic and human-made environment. Similarly, for older adults with local resident status, the statistically significant inverted U-shaped association between city population size and their self-rated health status emerges after controlling for cities’ socioeconomic and human-made environment. In contrast, for older adults without local resident status, this inverted U-shaped association becomes statistically insignificant after accounting for cities’ socioeconomic and human-made environment.

**Table 5:** Results of multilevel mixed-effects probit regression models stratified by individual characteristics.

	SRH (01)	SRH (01)	SRH (01)	SRH (01)	SRH (01)	SRH (01)
**Gender**	**Female**	**Male**	**Female**	**Male**	**Female**	**Male**
City population size	−0.004 (0.013)	0.016 (0.014)	0.129^*^ (0.075)	0.129 (0.079)	0.233^**^ (0.100)	0.221^**^ (0.105)
Quadratic term of city population size			−0.015^*^ (0.008)	−0.0123 (0.008)	−0.0301^**^ (0.013)	−0.027^**^ (0.013)
*N*	41,569	38,252	41,569	38,252	41,569	38,252
**Age**	**60–69**	**>69**	**60–69**	**>69**	**60–69**	**>69**
City population size	0.008 (0.015)	0.006 (0.015)	0.169^**^ (0.083)	0.064 (0.082)	0.249^**^ (0.108)	0.201^*^ (0.109)
Quadratic term of city population size			−0.0180^**^ (0.009)	−0.006 (0.009)	−0.0298^**^ (0.014)	−0.028^**^ (0.014)
*N*	48,097	31,724	48,097	31,724	48,097	31,724
**Educated status**	**Primary school and below**	**Junior high school and above**	**Primary school and below**	**Junior high school and above**	**Primary school and below**	**Junior high school and above**
City population size	0.002 (0.015)	0.006 (0.016)	0.096 (0.082)	0.149^*^ (0.088)	0.226^**^ (0.106)	0.192 (0.117)
Quadratic term of city population size			−0.011 (0.009)	−0.016^*^ (0.009)	−0.030^**^ (0.013)	−0.025^*^ (0.015)
*N*	34,327	45,494	34,327	45,494	34,327	45,494
**Living alone**	**Yes**	**No**	**Yes**	**No**	**Yes**	**No**
City population size	0.014 (0.021)	0.002 (0.014)	0.110 (0.076)	0.245^**^ (0.122)	0.367^**^ (0.165)	0.219^**^ (0.097)
Quadratic term of city population size			−0.012 (0.009)	−0.024^*^ (0.012)	−0.043^**^ (0.0200)	−0.029^**^ (0.012)
*N*	6,542	73,279	6,542	73,279	6,542	73,279
**Resident registration status**	**Yes**	**No**	**Yes**	**No**	**Yes**	**No**
City population size	0.002 (0.014)	−0.001 (0.021)	0.095 (0.079)	0.210^*^ (0.113)	0.241^**^ (0.101)	0.203 (0.149)
Quadratic term of city population size			−0.011 (0.009)	−0.023^*^ (0.012)	−0.032^**^ (0.013)	−0.027 (0.018)
*N*	59,249	20,496	59,249	20,496	59,249	20,496
**Individual characteristics**	Yes	Yes	Yes	Yes	Yes	Yes
**Cities’ socioeconomic and human-made environment**	No	No	No	No	Yes	Yes
**Cities’ geographical conditions**	Yes	Yes	Yes	Yes	Yes	Yes

(a) Standard errors in parentheses. (b)^*^ *p* < 0.1, ^**^ *p* < 0.05, ^***^ *p* < 0.01.

Building upon the outcomes of sorting analyses, we stratified the samples of older adults residing in cities with populations of less than 5 million and those with more than 5 million residents to assess disparities in the distribution of their self-rated health residuals based on individual characteristics (Table 6). Our findings reveal that, irrespective of gender or educational attainment (whether having received a junior high school education or higher), the distribution of self-rated health residuals among older adults in larger cities consistently exhibited positive left-truncation compared to those in smaller cities (Rows 1 and 2, Rows 5 and 6).

**Table 6:** Differences in the distribution of health residuals between smaller and larger cities across individual characteristics.

	Shift A	Dilation D	Truncation S	*N*	Pseudo R^2^
S_M_ to L_M_	−0.171^***^ (0.029)	0.938^**^ (0.029)	0.033^***^ (0.011)	38,252	0.653
S_F_ to L_F_	−0.190^***^ (0.015)	0.972^***^ (0.010)	0.020^***^ (0.004)	41,569	0.879
S_Y_ to L_Y_	−0.328^***^ (0.083)	0.862^**^ (0.067)	0.033^*^ (0.022)	48,097	0.720
S_O_ to L_O_	−0.158^***^ (0.011)	0.940^***^ (0.009)	−0.000 (0.001)	31,724	0.974
S_LE_ to L_LE_	−0.040^**^ (0.016)	0.976 (0.023)	0.021^**^ (0.010)	34,327	0.519
S_HE_ to L_HE_	−0.094^***^ (0.016)	0.990 (0.010)	0.027^***^ (0.003)	45,494	0.626
S_LT_ to L_LT_	−0.170^***^ (0.010)	0.964^***^ (0.008)	0.031^***^ (0.003)	73,279	0.732
S_LA_ to L_LA_	−0.229^***^ (0.030)	0.963^***^ (0.025)	0.005 (0.005)	6,542	0.978
S_NH_ to L_NH_	−0.159^***^ (0.013)	0.957^***^ (0.007)	0.034^***^ (0.003)	59,249	0.763
S_WH_ to L_WH_	−0.240^***^ (0.032)	0.926^***^ (0.024)	0.016 (0.008)	20,496	0.805
S_MS_ to L_MS_	−0.260^***^ (0.033)	0.886^***^ (0.025)	−0.000 (0.007)	12,987	0.825
S_ML_ to L_ML_	−0.269^***^ (0.032)	0.918^***^ (0.020)	0.027^***^ (0.007)	8,661	0.839

Standard errors are in parentheses. * *p* < 0.1, ** *p* < 0.05, *** *p* < 0.01. (a) S*: distribution of self-rated health residuals of older adults in cities of less than 5 million residents; L*: distribution of self-rated health residuals of older adults in cities of 5 million or more. (b) *M: distribution of self-rated health residuals of older male urban residents; *F: distribution of self-rated health residuals of older female urban residents. (c) *Y: distribution of self-rated health residuals of older adults aged below 70; *O: distribution of self-rated health residuals of older adults aged 70 and above. (d) *LE: distribution of self-rated health residuals of older adults with an education level of primary and below; *HE: distribution of self-rated health residuals of older adults with an education of junior high school and above. (e) *LT: distribution of self-rated health residuals of older adults living with families; *LA: distribution of self-rated health residuals of older adults living alone. (f) *NH: distribution of self-rated health residuals of older adults without local resident registration status; *WH: distribution of self-rated health residuals of older adults with local resident registration status. (g) *MS: distribution of self-rated health residuals of older adults who had left their places of resident registration less than ten years; *ML: distribution of self-rated health residuals of older adults who have left their places of resident registration over ten years.

The distributions of self-rated health residuals for older adults aged 70 and above, living alone, and those who left their places of resident registration for less than ten years did not exhibit left-truncation in larger cities relative to smaller ones (Rows 4, 8, 10, and 11). However, the distributions of self-rated health residuals for their counterparts exhibit significant left-truncation in larger cities relative to smaller ones (Rows 3, 7, 9, and 12). This inclination may be attributed to the greater mobility of younger older adults and the supportive environment provided by families, facilitating their mobility compared to older age groups and individuals living alone. Furthermore, older adults who left their place of resident registration but had not acquired local resident status in the destination for an extended period of time might choose to live in smaller cities when their health status deteriorates.

## 5 Discussion

The findings of this study underscore the significance of population size in influencing the self-rated health of older adults in urban areas. Notably, the relationship between a city’s population size and older adults’ self-rated health is revealed to be nonlinear, particularly when accounting for individual differences in socioeconomic status and variations in socioeconomic development levels across cities. This outcome aligns with broader studies encompassing diverse population groups and spanning cross-boundary analyses related to urbanization and public health (54). Moreover, our research highlights the impact of sorting decisions made by urban older adults based on their health status and unobservable factors. Older adults tend to gravitate towards environments that are socially and physically conducive to their well-being. In instances where unhealthy older adults encounter challenges accessing social security systems in large cities, choosing to reside in their hometowns or smaller cities becomes a rational decision, as these locales offer better social support.

### 5.1 Enhanced individual socioeconomic status, integrated within the broader socioeconomic development of cities, plays a crucial role in the health benefits attributed to the growth of city population size

Our findings reveal that older adults residing in cities with larger populations tend to exhibit better general health. However, this association transforms into an inverted-U shape, with a turning point around 1–1.5 million residents, when considering individual socioeconomic status. This suggests that the health benefits observed in cities with populations exceeding 1.5 million are primarily attributed to improvements in individual socioeconomic status. At the city level, when accounting for the Socioeconomic Development Index, we observe a more pronounced influence on altering turning points compared to controlling for other indicators such as the Environmental Pollution Index, Afforestation Index, and Pollution Abatement Index. These imply that the health benefits linked to population size growth are significantly influenced by both individual socioeconomic status and the overall socioeconomic development level of cities.

Concerning the association between city population size and the general health of older adults, the concave nature of the inverted U-shaped curve becomes more pronounced, or the turning point decreases, when accounting for the positive effects of urban population growth on health. Conversely, when considering the negative impacts, the turning point tends to increase, and the association leans towards a more linear or convex U-shaped curve. It is essential to acknowledge that the analysis of optimal population size holds more theoretical than practical value, providing insights into the underlying mechanisms that shape the association between city population size and older adults’ health. Moreover, several adverse factors, such as neighborhood conditions, social contacts, and living stress, could potentially confound the relationship between city population size and older adults’ self-rated health. Upon controlling for these factors, it is reasonable to anticipate a transition from the inverted U-shaped relationship to a more linear one. However, given the inherent challenges in observing or quantifying various community factors, further exploration is necessary to substantiate this trend.

### 5.2 Urban population growth has varied effects on health across different groups

Based on the analysis on the association between city population size and general health of various older groups, our findings suggest that certain characteristics significantly influence how older adults perceive the impact of the city’s population growth on their health. Older adults who are male, older, and have lower living standards and socioeconomic status—groups typically associated with poorer general health—tend to be more responsive to the compensatory effects of the improved socioeconomic and human-made environment in mitigating the risks associated with urban population growth. Conversely, their counterparts appear to be more sensitive to the unobserved influences associated with urban population growth. Moreover, the evolving socioeconomic and human-made environment resulting from urban population growth introduces health risks for migratory older adults but yields benefits for those with local resident status. These results align with previous studies that have focused on individual variations in health benefits derived from urbanization (55–57).

### 5.3 Health benefits in the cities with more than 5 million residents are overestimated as smaller cities host less-healthy older migrants

Urban expansion is generally anticipated to have a positive impact on the health of urban residents (58, 59). However, our sorting analyses, which account for the health-driven selection of residence, reveal that older adults with poorer health tend to choose living locations in cities with populations less than 5 million. This implies that the health benefits commonly attributed to population growth in cities with over 5 million residents might be overestimated. The sorting effects are more pronounced among older adults with greater resources supporting their mobility or those without a longstanding local resident status. In essence, older migrant adults with poorer health lean towards smaller cities, influenced in part by limited access to healthcare in large cities without obtaining local resident status. While large cities typically boast more healthcare resources, they also incur higher healthcare costs. This poses a challenging reality for older and/or unwell residents without local healthcare coverage. China’s segregated healthcare system particularly impacts those who lack wealth and reside outside their registered area. While healthier individuals may have more flexibility in their movements, this is not the case for those who require periodic healthcare assistance. In this study, a substantial 25.77% of respondents lacked local resident registration status, underscoring the noteworthy proportion of older adults who have relocated from their hometowns. Given that entitlement to public health services in China remains closely tied to resident registration, it is reasonable to expect that some respondents may need to return to their hometowns when healthcare becomes essential for them.

## 6 Conclusion

The population size of a city significantly influences the self-rated health of its older adults, with those residing in larger cities generally exhibiting better overall health. The study underscores the pivotal roles played by variations in individual socioeconomic status and the socioeconomic development level of cities in determining the health benefits linked to city population size. Moreover, empirical evidence highlights the impact of individual sorting decisions on the relationship between city population size and the self-rated health of older adults, revealing that cities with populations less than 5 million tend to attract less-healthy older migrants, leading to an overestimation of health benefits in cities with over 5 million residents. The study unveils distinct associations between city population size and the general health of diverse older groups, some of whom also make unique sorting decisions. Older adults who typically experience worse health due to their demographic, living, and socioeconomic status are more responsive to the compensatory effects of the improved socioeconomic and human-made environment, mitigating the risks associated with urban population growth. Moreover, the evolving socioeconomic and human-made environment resulting from urban population growth introduces health risks for migratory older adults but yields benefits for those with local resident status. The sorting effects are more pronounced among older adults with greater resources supporting their mobility or those without a longstanding local resident status.

To promote healthy aging, Chinese urban policymakers are encouraged to adapt planning and development strategies to consider the intricate relationship between city population size and the health of older adults. While promoting population agglomeration in small cities can yield health benefits for older adults, large cities, particularly those with populations exceeding 5 million, need to be vigilant about health risks associated with population growth among older adults. Imposing restrictions on settlement through the household registration system may improve the average health of older adults in large cities, but it may result in the departure of less-healthy older migrants rather than genuinely enhancing the health status of local older adults. Collaborating with surrounding small and medium-sized cities in older adults healthcare emerges as a viable option for large cities to mitigate built-environmental health risks and leverage their advantage in medical resources simultaneously. In summary, regardless of population size, Chinese cities should consistently prioritize the health of urban older adults when formulating and implementing urban policies.

## Data availability statement

The original contributions presented in the study are included in the article/Supplementary material, further inquiries can be directed to the corresponding author.

## Author contributions

ZP: Writing – original draft, Writing – review & editing. WD: Writing – review & editing. ZH: Writing – original draft, Writing – review & editing.

## Footnotes

^1^ https://microdata.stats.gov.cn/#/
