# Supplementary material for: Does the population size of a city matter to its older adults’ self-rated health? Results of China data analysis
Source: Front Public Health. 2024 Feb 1;12:1333961. doi: 10.3389/fpubh.2024.1333961 (PMC10867327; doi:10.3389/fpubh.2024.1333961)
Supplement: Supplementary file 1 [file Table_1.docx]

# Appendix

**Table A1 Rotated factor loadings (pattern matrix) of city-level covariates**

| Variable | Factor 1 | Factor 2 | Factor 3 | Factor 4 |
| --- | --- | --- | --- | --- |
|  | Socioeconomic Development | Environmental Pollution | Afforestation | Pollution Abatement |
| **Annual electricity consumption** | 0.9413 | 0.0294 | -0.07 | 0.1053 |
| **GDP** | 0.9754 | 0.0134 | 0.0006 | 0.061 |
| **Number of licensed (assistant) doctors** | 0.9367 | 0.1229 | 0.165 | -0.135 |
| **Urban built-up area** | 0.9198 | 0.072 | 0.1218 | -0.037 |
| **Average house price in 2015** | 0.8819 | -0.003 | 0.0736 | 0.024 |
| **Proportion of R&D funding in GDP** | 0.7657 | 0.0153 | 0.1527 | 0.0342 |
| **Coverage rate of pension for urban workers** | 0.7224 | -0.162 | 0.1891 | 0.4292 |
| **Coverage rate of medical insurance for urban workers** | 0.7232 | -0.127 | 0.1745 | 0.4402 |
| **Coverage rate of unemployment insurance for urban workers** | 0.6318 | 0.2077 | 0.0069 | -0.141 |
| **Average commuting time** | 0.6106 | -0.179 | 0.2652 | 0.4153 |
| **PM10** | -0.08 | 0.9546 | -0.007 | 0.0095 |
| **PM25** | 0.1653 | 0.9284 | 0.0339 | -0.008 |
| **CO** | -0.077 | 0.7386 | 0.0217 | -0.055 |
| **SO_2_** | -0.335 | 0.7044 | -0.027 | 0.1105 |
| **NO_2_** | 0.5033 | 0.6741 | 0.0561 | 0.2154 |
| **Green space and square area** | 0.1537 | 0.0559 | 0.9077 | 0.1 |
| **Coverage rate of greenbelt** | 0.1393 | 0.0295 | 0.9276 | 0.0449 |
| **Per capita park green area** | -0.083 | -0.05 | 0.6586 | -0.033 |
| **Harmless disposal of garbage per day (tons)** | -0.232 | -0.015 | -0.026 | 0.6235 |
| **Coverage rate of natural gas** | 0.2331 | 0.1902 | 0.3129 | 0.4706 |
| **Proportion of sewage disposal in sewage disposal plants** | 0.0566 | 0.2783 | 0.153 | 0.6165 |

Note: Factor 1, characterized by high correlations with annual electricity consumption, the number of licensed establishments, urban built-up area, average house price in 2015, proportion of R&D funding in GDP, coverage rate of pension for urban workers, coverage rate of medical insurance for urban workers, coverage rate of unemployment insurance for urban workers, and average commuting time, has been designated as the Socioeconomic Development Index. Factor 2, strongly associated with PM10, PM25, CO, SO2, and NO2, is identified as the Environmental Pollution Index. Factor 3, exhibiting high correlations with green space and square area, coverage rate of greenbelt, and per capita park green area, is named the Afforestation Index. Finally, Factor 4, marked by strong correlations with harmless disposal of garbage per day, coverage rate of natural gas, and the proportion of sewage disposal in sewage disposal plants, is referred to as the Pollution Abatement Index.
